# On the influence of discourse connectives on the predictions of humans and language models

**DOI:** 10.3389/fnhum.2024.1363120

**Published:** 2024-09-30

**Authors:** James Britton, Yan Cong, Yu-Yin Hsu, Emmanuele Chersoni, Philippe Blache

**Affiliations:** ^1^Department of Chinese and Bilingual Studies, The Hong Kong Polytechnic University, Hong Kong, China; ^2^School of Languages and Cultures, Purdue University, West Lafayette, IN, United States; ^3^LPL CNRS, Aix-Marseille University, Marseille, France

**Keywords:** discourse connectives, event knowledge, psycholinguistics, language models, Natural Language Processing

## Abstract

Psycholinguistic literature has consistently shown that humans rely on a rich and organized understanding of event knowledge to predict the forthcoming linguistic input during online sentence comprehension. We, the authors, expect sentences to maintain coherence with the preceding context, making congruent sentence sequences easier to process than incongruent ones. It is widely known that discourse relations between sentences (e.g., temporal, contingency, comparison) are generally made explicit through specific particles, known as *discourse connectives*, (e.g., *and, but, because, after*). However, some relations that are easily accessible to the speakers, given their event knowledge, can also be left implicit. The goal of this paper is to investigate the importance of discourse connectives in the prediction of events in human language processing and pretrained language models, with a specific focus on concessives and contrastives, which signal to comprehenders that their event-related predictions have to be *reversed*. Inspired by previous work, we built a comprehensive set of story stimuli in Italian and Mandarin Chinese that differ in the plausibility and coherence of the situation being described and the presence or absence of a discourse connective. We collected plausibility judgments and reading times from native speakers for the stimuli. Moreover, we correlated the results of the experiments with the predictions given by computational modeling, using Surprisal scores obtained via Transformer-based language models. The human judgements were collected using a seven-point Likert scale and analyzed using cumulative link mixed modeling (CLMM), while the human reading times and language model surprisal scores were analyzed using linear mixed effects regression (LMER). We found that Chinese NLMs are sensitive to plausibility and connectives, although they struggle to reproduce expectation reversal effects due to a connective changing the plausibility of a given scenario; Italian results are even less aligned with human data, with no effects of either plausibility and connectives on Surprisal.

## 1 Introduction

According to psychologists and cognitive scientists, language understanding requires the construction of a dynamic mental representation of the state of affairs denoted by a text (Van Dijk and Kintsch, 1983; Zwaan, 2016). A commonly-used notion is the one of *situation models*, data structures containing a representation of the event that is currently being processed/understood (Zwaan and Radvansky, 1998). The comprehension process takes place within an existing situation model, and the model is dynamically and incrementally updated by unifying the current content with the new information coming in. At the same time, psycholinguistic research brought evidence that human semantic memory stores a generalized knowledge about events and their participants (McRae et al., 1998; Ferretti et al., 2001; McRae et al., 2005; Hare et al., 2009; McRae and Matsuki, 2009). Humans quickly activate this knowledge to anticipate upcoming input while understanding texts, and the coherence of the actual input with the expectations affects processing complexity: for example, sentences including highly-predictable verb-argument combinations are associated with shorter reading times, shorter eye fixations, and reduced N400 amplitudes in ERP studies,^1^ compared to sentences with more unexpected and “surprising” event participants (e.g., *The journalist checked the spelling* is read faster than *The journalist checked the brakes*, as *brakes* is more unlikely as a patient in the second sentence) (Bicknell et al., 2010; Matsuki et al., 2011). It has been previously suggested that the extra processing difficulty may be due to the cost of unifying in the situation model portions of the event knowledge that have been activated by the linguistic input but have a low degree of semantic plausibility (Chersoni et al., 2016, 2017, 2021b).

Reading times and N400 effects have also been shown to depend on the wider discourse context, and not just on the verb-argument relations within the sentence. For example, words that are acceptable in a local context but are anomalous in the general discourse lead to longer reading times and larger N400 effects (Van Berkum et al., 1999, 2005). However, a text may be explicitly signaling that the upcoming propositions are unexpected or contradictory given what was said before. From this point of view, *discourse connectives* (e.g., *but, although, because, therefore* etc.) play an important role in indicating the semantic relation between text spans (Danlos et al., 2018); they can be used by speakers to increase the coherence of the texts, helping listeners at the same time to update their situation models, thus modulating their expectations about what could be the plausible upcoming words.

To this latter goal, the most interesting connectives are those expressing *opposition relations*, i.e., concessive and contrastive connectives (Izutsu, 2008). According to Lakoff (1971), contrastive connectives indicate a direct semantic opposition between two clauses (e.g., *The weather today is sunny*, ***but*** *yesterday it was rainy*), while concessive connectives inform the listener that a given expectation has been denied (e.g., *She works as a lawyer for Sullivan & Co, but she is not a bad person*, implying that the speaker generally has a negative opinion about the lawyers working for that firm). It is easy to see why connectives are important in articulating the grammar of the discourse and facilitate sentence processing: when the listeners should *revert* their expectations about what is coming next, such particles can inform them about the necessity to update their situation model accordingly. On the other hand, it should be kept in mind that there is no one-to-one mapping between connectives and discourse relations (Knott, 1996), and their interpretation is probabilistic (Asr and Demberg, 2020), i.e., depending on the distribution of relations that a connective has been used to signal in one's own linguistic experience. In our previous examples, it can be noticed indeed that *but* can support either a contrastive and a concessive meaning. There might be some margin of subjectivity in the interpretation of a discourse connective, especially when the context does not provide strong disambiguation cues, and the odds of a given discourse relation might be different between the connectives of the same type across languages.

In the recent research in Natural Language Processing (NLP), the field of Artificial Intelligence that is concerned with giving machines the ability to generate and understand human language, a new class of **neural language models** (henceforth NLMs) has emerged as the dominant one in the literature. Such models are generally based on the Transformer architecture (Vaswani et al., 2017) and are able to generate rich and dynamic representations of words in context, leading to remarkable improvements in several supervised linguistic tasks (Devlin et al., 2019; Liu Y. et al., 2019; Yang et al., 2019). Over the last few years, with the increase of architectural complexity and the amount of training text, NLMs are more and more often evaluated in a zero-shot fashion, by letting them freely generate the answers to the task instances (Radford et al., 2019; Brown et al., 2020; Achiam et al., 2023).

NLMs are trained on the task of predicting the next word given a sequence of words,^2^ and one of the reasons for their success is that they can be *pre-trained* without the need of annotated data, as the objective of the network is to reproduce the original word sequences in a large corpus of text (*self-supervised learning*, see Manning et al., 2020). The pretraining phase allows the models to encode a lot of knowledge about (the statistical patterns of) language in their internal representations. In this sense, the usage of the distributional behavior of linguistic expressions in NLMs as a way to represent their meaning could be seen in full continuity with the tradition of Distributional Semantics (Lenci, 2023).

Another common evaluation of NLMs makes use of their log-probability or **Surprisal** (the negative log of the probability) scores to account for a wide range of sentence processing phenomena (Futrell et al., 2018; Van Schijndel and Linzen, 2018; Wilcox et al., 2018), including facilitation (Michaelov and Bergen, 2020, 2022a,b; Michaelov et al., 2023) and interference (Ryu and Lewis, 2021; Cong et al., 2023b) effects in online sentence processing.^3^ The idea behind Surprisal is that words that are less predictable should take more time for humans to process, and this predictability can be estimated via a NLM. In psycholinguistics, the predictability of a target word is often manipulated in order to differ by experimental condition. Therefore, the goal of modeling studies is to see whether the scores estimated by a NLM align with human behavioral results.

The goal of the present paper is to analyze, across different languages, the processing effect associated with discourse connectives reflecting opposition relations, in two different perspectives: human perception and computational prediction. We are specifically interested in concessives because of their special effects on event-based expectations: a comprehender generally makes a prediction expecting a plausible, coherent scenario to unfold, but a concessive connective signals that such expectations are going to be reversed. Therefore, we want first to see if we can replicate the findings of Xiang and Kuperberg (2015) on concessives at the behavioral level in Italian and Mandarin Chinese, to verify if we observe the same effects in two new languages. Furthermore, we also introduce a condition with contrastive connectives to see if they behave similarly. Lastly, we want to test if NLMs can keep track of the expectation reversal, and whether their Surprisal scores reflect the update in the situation described by the discourse. Concretely, on the basis of the story stimuli of a dataset introduced by Xiang and Kuperberg (2015), we first built similar datasets for Italian and Mandarin Chinese and we collected judgements about the plausibility of the events in the stories from native speakers; then, we collected reading times in a self-paced reading experiment in both languages. We observed that the two languages exhibit distinct patterns, both in terms of plausibility ratings and of self-paced reading times.

Next, we computed the Surprisal scores for the target verbs in the experimental stimuli using the GPT-2 language models^4^ for Italian and Mandarin Chinese, in order to observe the extent to which they were affected by the general plausibility of the stories and by the presence of discourse connectives. We found that NLMs do not reproduce the same effects observed in human data, in particular in Italian. We speculated that this could be due either to the relatively small size of the NLM used in our experiments, or to a large percentage of target words in our datasets that are not included in the models' vocabulary. Our analysis of the results suggested that the latter could be the most important factor.

## 2 Related work

### 2.1 Discourse coherence and connectives in sentence comprehension

In natural language, individual sentences are generally preceded by a broader discourse context. Scholars since Zwaan and Radvansky (1998) have argued that humans use situation models to form an event-based representation of what is being communicated, and that such representations are continuously updated as new input comes in. Coherence in discourse notoriously facilitates human language processing, as shown by experimental studies using different methodologies, e.g., self-paced reading (Albrecht and O'Brien, 1993), naming tasks (Hess et al., 1995), eye-tracking and ERPs (Camblin et al., 2007). In other words, as long as the new information is coherent with the event knowledge, the comprehender can easily integrate it into the current situation model.

Kuperberg (2013) distinguishes three layers in human event representations: (i) the layer of *event sequences*, related to our knowledge about the likely temporal, spatial and causal connections linking event and states together to form sequences of events, also known as scripts (Schank and Abelson, 1975); (ii) the layer of *event structures*, corresponding to our knowledge of events and their typical participants (cf. the notion of “generalized event knowledge” in McRae and Matsuki, 2009); (iii) the layer of *semantic features*, concerning our knowledge of the features and properties of conceptual entities and categories. Xiang and Kuperberg (2015) argue that discourse connectives influence primarily the representation of event sequences: for example, when a comprehender is processing a discourse and then hears or reads a concessive connective (e.g., *even so*), his or her predictions about the upcoming event will be reversed, as the connective will be signaling to expect an opposite-to-expected causal relationship.

In their experiment, Xiang and Kuperberg (2015) designed a set of 3-sentence stories in four different conditions, differing in degree of coherence of the last sentence with the discourse context and for the presence or not of the *even so* connective at the beginning of the last sentence. After collecting coherence judgements from humans, they found that no-connective coherent items had the highest coherence ratings, whereas the no-connective incoherent items had the lowest ones. The coherent items with an *even so* connective (e.g., *Liz had a history exam on Monday. She took the test and failed it. Even so, she went home and celebrated wildly*). In a follow-up ERP experiment, measuring the N400 amplitude at the main verb in the final sentence (e.g., *celebrated*, in the example above), they found that the N400 for the verb was more reduced in the coherent *even-so* items (i.e., lower processing costs), compared to the plain coherent items, suggesting that the presence of the connective made the prediction of the verb even easier.

The data by Xiang and Kuperberg (2015) exemplify discourse expectations in a relatively short range, driven by linguistic elements within the same sentence or in an adjacent sentence. Their work was extended by Scholman et al. (2017), who focused on the discourse markers *on the one hand… on the other hand…* to check whether comprehenders can maintain discourse coherence expectations across multiple sentences. They setup a norming experiment, where the subjects where exposed to stories introduced by *on the one hand*, followed by other propositions introduced by connectives, including contrastive ones, and finally by a proposition introduced by *on the other hand*. The results showed that subjects were keeping track of all the embedded constituents and their dependencies, and that they dispreferred stories with situations of contrast introduced by *on the other hand* if the previous context already included a contrast with the situation introduced by *on the one hand*.

Köhne-Fuetterer et al. (2021) presented experiments with visual world paradigm and ERPs in English and German, using story items containing causal or concessive connectives. The visual world experiment revealed that the anticipatory looks toward a target object depended on the specific discourse connective in the item. Moreover, in the ERP experiment the authors found a late positivity on the concessive connectives compared to causal ones in both languages, possibly reflecting the extra processing costs of expectation reversal.

It is not easy to understand to what extent the connective and coherence-driven effects transfer to other languages. Most of the current studies were focusing on English, and in some cases, on German (e.g., the visual world and ERP study by Köhne-Fuetterer et al., 2021). Even in English, the interpretation of connectives has been claimed to be probabilistic, and individual differences can be observed in the expecting coherence relations (Asr and Demberg, 2020; Scholman et al., 2020). It is also worth mentioning that recent research established that the usage itself of predictive processing in sentence comprehension is flexible, heavily depending on the reliability of the prediction, and it can be “dismissed” when a discourse is not coherent. For example, Brothers et al. (2019) presented an ERP study in which subjects heard sentences from different speakers, but one speaker was instructed to frequently produce completions that violated the expectations of the listeners (“unreliable speaker”). The authors found that reliability affected N400 amplitudes, with larger effects of predictability when the speaker was reliable, as if the subjects were less engaging in predictions after seeing that those predictions were often violated. In the same line of work, the self-paced reading experiments by Carter and Hoffman (2024) showed that the general coherence of discourse is an important factor; comprehenders are sensitive to shifts in the topics and they tend to “predict less” when they face incoherent discourse structures.

### 2.2 NLM Surprisal for modeling sentence processing data

Transformer-based NLMs (Vaswani et al., 2017; Devlin et al., 2019; Radford et al., 2019) have become increasingly popular in NLP research, and a growing body of literature aims at investigating the kind of linguistic knowledge they encode, and to what extent they can reproduce human performance in language processing tasks. A common methodology is the so-called *probing* (see, *inter alia*, Tenney et al., 2019a,b; Jawahar et al., 2019; Hewitt and Liang, 2019; Liu N. F. et al., 2019; Wu et al., 2020; Vulić et al., 2020; Sorodoc et al., 2020; Koto et al., 2021; Chersoni et al., 2021a; Thrush et al., 2022). In this methodology, a relatively simple classifier is asked to solve a linguistic task (e.g., number agreement, anaphora agreement etc.) using a representation derived from a NLM without any specific linguistic supervision. If the classifier succeeds, then the NLM can be inferred to encode the target linguistic knowledge in its representations.

Other studies focused directly on the **Surprisal** scores computed by the models, to understand the extent to which they are sensitive to linguistic phenomena that have been shown to affect human sentence processing. The Surprisal of a word is a measure of its predictability given the previous context (Hale, 2001; Levy, 2008). It is defined as the negative logarithm of the probability of the word given the context and it is generally correlated with human reading times (i.e., more surprising words are read more slowly by humans). For example, a work by Misra et al. (2020) investigated the predictions of BERT in a setting aimed at reproducing human semantic priming; they reported that BERT (Devlin et al., 2019) was indeed sensitive to “priming”, in a way that it predicted a word with lower Surprisal values when the context included a related word as opposed to an unrelated one. Cho et al. (2021) modeled a priming effect on the prediction of typical event locations, which was observed in humans to be related to the imperfective aspect of the verb. The authors found that BERT outputs lower Surprisal scores for typical locations, but differently from humans, and it manages to do so regardless of the aspect of the main verb.

Michaelov and Bergen (2022a) used Surprisal to investigate the issue of collateral facilitation, that is, a scenario when anomalous words in a sentence are processed more easily by humans due to the presence of semantically related words in the context. They compared the scores obtained from several Transformer NLMs with human data from several sentence processing experiments, and found that most of the models reproduced the same significant differences between conditions that were observed for humans' behaviors. In Michaelov et al. (2023), Surprisal was utilized instead to replicate the effect of the discourse context in reducing the N400 amplitude for anomalous words, using the Dutch stimuli in the experiments by Nieuwland and Van Berkum (2006) as the evaluation data. In such experiments, Nieuwland and Van Berkum (2006) showed that sentences containing verb-object animacy violations (e.g., *The girl comforted the clock*) elicited large N400 effects, but the inclusion of a supportive discourse context (e.g., a girl talking to a clock about its depression) lead to reduction of this effect. Language models showed, once again, a very close pattern to humans, suggesting that the reduction effect of the original study may be due to lexical priming from the previous context.

### 2.3 Discourse connectives in NLP

The importance of connectives in NLP research is due to the fact that they lexicalize specific discourse relations (Braud and Denis, 2016; Ma et al., 2019). During the acquisition of annotations for discourse-parsing tasks, the connectives sometimes provide a clue to the discourse relations, which are sometimes implicit. In such cases, human annotators are asked to insert the connective that they consider to be more appropriate, given two discourse fragments (Prasad et al., 2007). Given the recent rise of NLMs, researchers in NLP started to explore the capacity of the models to identify the right connectives, which requires in turn an understanding of the relations between discourse fragments.

Ko and Li (2020) proposed to investigate GPT-2's linguistic competence in terms of discourse coherence by testing the model's ability to produce the correct connectives, when given a discourse relation linking two clauses. Using both organic generation and fine-tuned scenarios, they observed that GPT-2 did not always generate coherent discourse, although the generations were better aligned with human behavior in the fine-tuned scenario.

Pandia et al. (2021) evaluated several NLMs on the prediction of the correct connectives in contexts that required Gricean-like pragmatic knowledge and in which a specific connective would correspond to an implicature. For example, in cases such as *Maggie did the paperwork by hand* ***and*** *the company bought new computers, which is to say, Maggie did the paperwork by hand [MASK] the company bought new computers*., the model had to predict *before* in the [MASK] position to show an understanding that the implied meaning of *and* in this context was *and then*. The authors showed that, when controlling strictly for low-level lexical and syntactic cues, the models performed at chance level at best.

The recent work of Cong et al. (2023a) is the closest one to our study, since it investigates the impact of discourse connectives on the Surprisal scores of the NLMs. The authors analyzed the effects of concessive and contrastive connectives on NLMs with the English stimuli by Xiang and Kuperberg (2015), by measuring the Surprisal scores of the target verbs in their stories in different experimental conditions. They tested several NLMs of different size (e.g., GPT-2 Base, GPT-2 XL and GPTNeo) and found that the larger GPTNeo model was the one showing a pattern closer to human behavior when a concessive connective was used, leading to a reversal of the expectations on the final verb. Moreover, the results were still consistent after replacing the original *even so* with different concessive connectives. On the other hand, and according to the prediction of linguistic theory (Izutsu, 2008), they found that replacing a concessive with a contrastive connective does not lead to expectation reversal effects.

Our study expands on the previous work of Cong et al. (2023a). First of all, we test whether expectation reversal can also be observed in other languages - we ran our experiments in Italian and Mandarin Chinese, two typologically different languages from English (the former a romance language, the latter a sinitic language). We collect Italian and Chinese native speakers' coherence judgments and self-paced reading of the same items translated from items used in Xiang and Kuperberg (2015). Then we model the same experimental items with each language's GPT-2 Base model.

## 3 Behavioral experiments

Concessive connectives can be used to create scenarios where the expectations have to be reversed. In translating the English dataset used in Xiang and Kuperberg (2015), we aimed at using connectives that prototypically represent concessives in the target languages, to closely reproduce the original stimuli. We collected human ratings on the naturalness and plausibility of the situations described by our experimental items, and unlike the original study, we tested human processing behavior using a self-paced reading task.

In addition to items with concessive connectives based on the original study, we created an additional version of our stimuli using *contrastive* connectives. Although contrastive connectives *per se* simply signal a contrast, rather than the denial of expectations, they can still be used in concessive constructions. While using a contrastive connective for a concessive relation may not be prototypical, it should still be understandable to the readers, as the interpretation of connectives is probabilistic (Asr and Demberg, 2020). Moreover, for our purposes, the use of two types of connectives helps examine the influence of connective types on human comprehension of concessive scenarios.

### 3.1 Experimental items

We used the datasets created by Xiang and Kuperberg (2015) as our starting point to develop similar datasets for Italian and Mandarin Chinese. In their experiment, the authors designed 180 sets of three-sentence discourse items in English, each with four conditions as in Example 1 (45 scenarios per condition). The target word (underlined) was always the main verb of the final sentence.

(1) a. Liz had a history exam on Monday. She took the test and **aced** it. She went home and *celebrated* wildly. (*Plain, Coherent*)
b. Liz had a history exam on Monday. She took the test and **failed** it. She went home and *celebrated* wildly. (*Plain, Incoherent*)
c. Liz had a history exam on Monday. She took the test and **failed** it. *Even so*, she went home and *celebrated* wildly. (*Even so, Coherent*)
d. Liz had a history exam on Monday. She took the test and **aced** it. *Even so*, she went home and *celebrated* wildly. (*Even so, Incoherent*)

Notice that in conditions (1c) and (1d), the presence of the connective *even so* changes the general coherence of the given scenario, reversing the expectations of the reader. In our work, we decided to refer to the notion of *plausibility* instead of coherence, in order to focus more on the plausibility/naturalness of the described scenario rather than on the discourse connections. We believe plausibility will be a more intuitive notion to grasp for the human readers. Therefore, in this paper, we refer to condition like (1a) as *Plaus*, (1b) as *Implaus*, (1c) as *ES-Plaus*, and (1d) as *ES-Implaus*.

Compared to Xiang and Kuperberg (2015), we have two additional conditions where the concessive connective in (1c) and (1d) is replaced by a contrastive one, as an example shown in Table 1. We refer to the contrastive conditions in each language dataset as *HW-Plaus* for a more plausible story containing a contrastive connective, and *HW-Implaus* for a less plausible story containing a contrastive connective. We translated *even so* to 即使如此 in Chinese and to *Nonostante ció* in Italian, and used contrastive connectives 但是 for Chinese and *tuttavia* for Italian, respectively; both translatable as *however* in the target languages (Battaglia and Pernicone, 1985; Wang, 2011).

**Table 1:** Example of the connective dataset item in all the conditions for each language with translation.

**Condition**	**Italian**	**Mandarin Chinese**
(a) Plain Implaus	La donna cieca era stata operata parecchie volte. Il dottore era molto deluso dai risultati. La sua vista improvvisamente migliorò quella notte.	失明的/妇女/接受/了/手术。/医生/对/手术/结果/表示/悲观。/妇女/的/视力/问题/改善/了/很多。
English translation	“The blind woman had undergone several operations. The doctor was very disappointed with the results. His visit that night brought a sudden improvement.”	
(b) Plain Plaus	La donna cieca era stata operata parecchie volte. Il dottore era molto ottimista sui risultati. La sua vista improvvisamente migliorò quella notte.	失明的/妇女/接受/了/手术。/医生/对/手术/结果/表示/乐观。/妇女/的/视力/问题/改善/了/很多。
English translation	“The blind woman had undergone several operations. The doctor was very optimistic about the results. His visit that night brought a (sudden) improvement.”	
(c) ES-/HW- Plaus	La donna cieca era stata operata parecchie volte. Il dottore era molto deluso dai risultati. {Nonostante ciò;Tuttavia}, la sua vista improvvisamente migliorò quella notte.	失明的/妇女/接受/了/手术。/医生/对/手术/结果/表示/悲观。/ {即使如此; 但是}/妇女/视力/问题/改善/了/很多。
English translation	“The blind woman had undergone several operations. The doctor was very disappointed with the results. {Even so; However}, the woman's vision suddenly improved that night.”	
(d) ES-/HW- Implaus	La donna cieca era stata operata parecchie volte. Il dottore era molto ottimista sui risultati. {Nonostante ciò; Tuttavia} la sua vista improvvisamente migliorò quella notte.	失明的/妇女/接受/了/手术。/医生/对/手术/结果/表示/乐观。/ {即使如此; 但是}/妇女/视力/问题/改善/了/很多。
English translation	“The blind woman had undergone several operations. The doctor was very optimistic about the results. {Even so; However}, the woman's vision suddenly improved that night.”	

Slashes indicate words in Mandarin sentences; connectives are underlined.

The stories of the original dataset were initially translated into the new languages by using the DeepL translation software.^5^ The sentences were manually checked one-by-one by two native speakers of Italian and Mandarin, to ensure the naturalness and coherence contrast of the translations in the new language datasets, and to correct possible mistakes. For both target languages, at least one of the authors is a native speaker. Stories for which it was not possible to achieve a natural-sounding translation were revised or excluded from the dataset. In the end, we came up with 100 stories for Italian and 180 stories for Mandarin Chinese for each of the three connective conditions (no connective, concessive, and contrastive). In both languages, each story has three clauses. For the Mandarin set, all sentences were adjusted to be 18 words in length, and in the Italian set all sentences were 22 words. The connective is always the first word of the third clause (Region 12 in Mandarin and Region 16 in Italian), and the target word is the main verb of the third clause that changes the potential plausibility of a given story. We ensured that no additional connectives occurred among the clauses in each story.

To prepare for the self-paced reading task, we split the whole dataset of each language (3 sentence types × 2 plausibility × 50 items in Italian, and 3 sentence types x 2 plausibility x 90 items in Chinese) by the Latin Square design so that each list has all the connective conditions (examples for each language and condition can be seen in Table 1).

### 3.2 Experiments

This section reports the findings of experiments with human participants.

#### 3.2.1 Participants

138 Mandarin native speakers (mean age: 23.00, SD: 3.92; 61 females) and 133 Italian native speakers (mean age: 28.95, SD: 5.21; 70 females), who did not report having any left-handedness nor family history of brain damage or speech or hearing impairment, were invited via online questionnaires. All received an explanation of the study and its procedures and gave their informed consent before its commencement. These participants were then directed to the online experiment in a self-paced reading format, which was administered at PCIbex (farm.pcibex.net). Participants were paid 100 HKD upon successfully completing the experiment.

#### 3.2.2 Self-paced reading experiment and ratings

During the experimental session, each participant was instructed to make sure the Internet connection was stable and to sit in front of a computer with a proper screen display of the online page of the experiment. The experimental material incorporated a “click-to-proceed” element that prevented the participants from reading ahead, and from understanding any whole story until they had finished reading it. Using this method, it was possible to isolate their reactions to specific regions within each story.

Each story was presented on screen one word at a time, in a non-cumulative, centered-window, self-paced reading paradigm (Just et al., 1982). The participant presses a key to mask the current word and reveal the next word. Upon completing four practice stories, each participant was asked to read the experimental stories carefully but at their natural reading pace. At the end of each story, the participant was asked to provide a rating of the level of plausibility and naturalness of the story they just read on a Likert scale from 1 (not plausible at all) to 7 (very plausible). The whole procedure took each participant between 35 and 45 min to complete.

The participants' reading times for each word and the rating time (i.e., the choice) were recorded to allow us to estimate the processing effort of reading comprehension (Just et al., 1982). Participants' ratings were also recorded for each item. Data points were excluded if the same rating was selected 10 or more times consecutively or only used two of the possible seven points on the scale.

#### 3.2.3 Analysis

We first excluded the data from participants who had abnormally fast or slow completion times (2.5 SD from the mean reading time), and then further recruitment was carried out to make sure each of the eight lists had at least 15 valid participants' data. After the data exclusion, 120 Mandarin speakers' and 133 Italian speakers' data were kept for the final analyses.

Then the reading times (RTs) were log-transformed to approximate a normal distribution for analysis purposes. For RTs, we analyze the overall RTs of each item, the target regions (i.e., the connective region, the keyword region and the end of the final sentence region) as well as the time participants used to provide ratings (i.e., the choice). RTs were fitted through generalized linear mixed effect models, with the predictors being condition and plausibility, and the random effects included the IDs of participants and items. As an additional predictor, we used the logarithmic frequency of the target word, which was extracted with the wordfreq Python tool (Speer, 2022).^6^ Pairwise *post-hoc* Tukey's comparisons were then conducted with R's emmeans package.

For the plausibility ratings, we analyzed the impact of conditions and plausibility using cumulative link mixed models produced by the clmm() function of the ordinal package (Christensen, 2023) in R. Pairwise *post-hoc* Tukey's comparisons were then conducted with R's emmeans package.

### 3.3 Results and analyses

**Figure 1:**
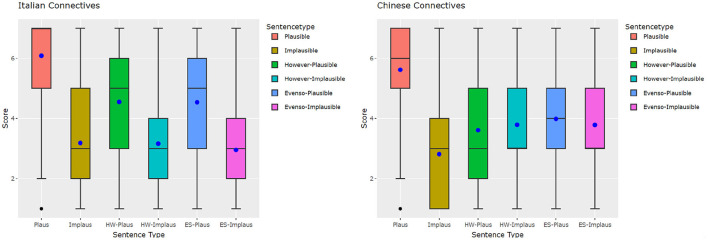
Italian and Chinese human plausibility judgments plotted by condition. Dot, mean; inner box line, median.

Based on the boxplots for Italian (left) and Chinese (right) human ratings in Figure 1, both language groups rated the plausible condition without connective the highest. The median rates in the Italian group show that implausible items with connectives are rated similarly to the respective no connective conditions in Italian, but the means (the dots) of plausible items with connectives are much lower than the plausible plain ones. In the implausible conditions, the mean ratings of *even so* are slightly lower than the other two implausible conditions.

In the Chinese group, the median and the mean ratings of plausible items with connectives are much lower than in the plausible condition without connectives, similarly to Italian. However, among the implausible conditions, while the median ratings are almost the same, the mean ratings of HW- and ES-Implaus are higher than the Implausible condition, and are close to the mean ratings of their plausible counterparts.

We fitted the ratings in CLMM with a full model, where connective and plausibility were the main effects, and their interaction was also included. Participant and item were included as the random effects. Results of the CLMMs were significant for all main effects (*p* < 0.01) for both Italian and Chinese. *Post-hoc* Tukey comparison was carried out using the emmeans() function from the emmeans library in R (Lenth, 2024). Here, we focus on the interaction effects' results. For the Italian set, there were significant contrasts (*p*s < 0.001) for all comparisons **except**:

- evenso-plausible/however-plausible (*p* = 1.0000)
- evenso-implausible/however-implausible (*p* = 0.3550)
- null-implausible/evenso-implausible (*p* = 0.4982)
- null-implausible/however-implausible (*p* = 0.9953)

For the Chinese set, there were significant contrasts (*p*s < 0.001) for all comparisons **except**:

- evenso-plausible/however-plausible (*p* = 0.1017)
- evenso-implausible/however-implausible (*p* = 0.9627)

In summary, for both Italian and Chinese, there were no significant differences in the ratings between the two connective types for both plausible and implausible conditions. However, the two language groups still show differences. For the Italian data, no significant differences were found between the implausible condition with no connective (null) and the two connective implausible conditions (“however”: *p* = 0.9953, “even so”: *p* = 0.4982). In contrast, the Chinese data **did** show a difference in that the no connective implausible condition was rated significantly lower than both connective conditions (*p* < 0.01).

As noted above, one important difference observable in the plots in Figure 1 is that all the Italian conditions show a difference between plausible and implausible, but in Chinese, the presence of a connective appears to even out the differences in plausibility, so that there is not as clear a distinction between plausible and implausible. For example, although still significant, the magnitude of the effect for the contrast between however-implausible/however-plausible (*p* < 0.05) is smaller than for all the other contrasts (see also the green and the light blue boxplot in Figure 1, right).

It is noticeable that, compared to the no connective implausible condition, the participants rated the plausible conditions with connectives higher. This is an effect of expectation reversal: without the connective, the situations described by *even so/however* plausible would be implausible; but since the connective is signaling that the last sentence will “contradict” the expected scenario, the entire story sounds more coherent as a whole (e.g., compare *Jane failed the test. She celebrated wildly*. with *Jane failed the test*. ***However/Even so***, *she celebrated wildly*.).

#### 3.3.1 Reading times

We analyzed and reported the reaction time of two critical regions recorded from the self-paced reading studies, i.e., the region of the target word (the main verb of the third clause) and the region of the end of the third sentence. Before the target word, we also measured the effect of sentence type in the connective region (no connective, *even so* or *however*), but not the other effects because before the connective position there is no difference between the items across conditions. We used *lmerTest* (Kuznetsova et al., 2017) and not the more standard *lmer* because only the former is able to return *p*-values for models with random effects.

For the connective region, *even so* conditions produced significantly slower reading times in both Italian (*p* < 0.05) and Chinese (*p* < 0.001), but the *however* condition was only significantly slower in Italian (*p* < 0.01) and not in Chinese (*p* = 0.05).

**Figure 2:**
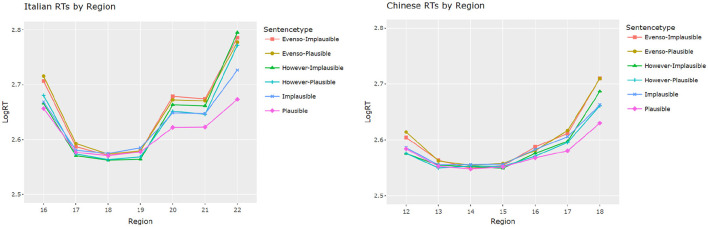
Log Reading Times per region. *y*-axis, reading time (ms); *x*-axis, sentence region.

Figure 2 shows the reading time (RT) of each word in the third clause of Italian (left) and Chinese (right) data by conditions of connective and plausibility, and the statistical analysis with the linear mixed effects models are available in Tables 2, 3. In Table 2 we can see that, in the target verb region, *however* items were read longer than the no connective condition in Italian (*p* < 0.001), but there was no significant difference for Chinese (*p* = 0.91). Additionally, in Italian we can see that the subjects took longer for the *even so* connective (*p* < 0.001) condition, and were slower in reading implausible items (*p* < 0.01). In Chinese we have a similarly strong effect of *even so* (*p* < 0.001). No significant effect was observed for frequency and plausibility in Chinese for the target word region, and more in general, the increase in reading times due to the *even so* connective is the only significant effect observed for Chinese in this region. Finally, in Italian we also see a facilitatory effect of the frequency of the target (*p* < 0.001).

**Table 2:** Results of Italian and Chinese reading times (RTs) for the target word region.

	**Italian**			**Chinese**		
	***Est***.	**SE**	***p***	***Est***.	**SE**	***p***
Intercept	67.92	34.96	0.05	81.27	61.45	0.19
ConnectiveE	79.90	19.46	< 0.001^***^	18.07	5.08	< 0.001^***^
ConnectiveH	69.10	19.45	< 0.001^***^	0.60	5.08	0.91
Implausible	43.37	15.88	< 0.01^**^	5.89	3.85	0.13
Freq	−32.19	6.93	< 0.001^***^	−10.33	13.89	0.46
ConnectiveE:implausible	−11.43	27.52	0.68	−0.43	6.66	0.95
ConnectiveH:implausible	−24.82	27.52	0.36	−7.21	6.66	0.28

SE, standard error; Freq, log frequency; RTs were recorded in milliseconds and centered for analysis. Significance levels of *p* values ^*^p < 0.05, ^**^p < 0.01, ^***^p < 0.001.

**Table 3:** Results of Italian and Chinese reading times (RTs) for the end of sentence region.

	**Italian**			**Chinese**		
	***Est***.	**SE**	***p***	***Est***.	**SE**	***p***
Intercept	60.63	50.10	< 0.05^*^	−262.53	100.87	< 0.05^*^
ConnectiveE	201.72	28.24	< 0.001^***^	190.24	8.89	< 0.001^***^
ConnectiveH	218.22	28.21	< 0.001^***^	89.87	8.16	< 0.001^***^
Implausible	88.69	23.04	< 0.001^***^	65.04	6.42	< 0.001^***^
Freq	−53.41	10.05	< 0.001^***^	42.06	22.44	0.06
ConnectiveE:implausible	−49.67	39.92	0.21	−28.81	11.53	< 0.05^*^
ConnectiveH:implausible	−26.27	39.91	0.51	−31.88	10.78	< 0.01^**^

SE, standard error; Freq, log frequency; RTs were recorded in milliseconds and centered for analysis. Significance levels of *p* values ^*^p < 0.05, ^**^p < 0.01, ^***^p < 0.001.

Considering the potential spillover effects, we also ran analyses of RTs at the end region of each condition (Table 3). Both languages showed significant effects for plausibility on RTs (*p* < 0.001). While no distinction between plausible and implausible was seen in Chinese at the target word region, significantly increased RTs for implausible items were observed at the end of sentence region (*p* < 0.001).

Moreover, stories containing a connective were processed significantly longer than the baseline condition (*p*s < 0.001), and this time regardless of the connective, as in both Italian and Chinese we can see that the *even so* and the *however* items are read significantly slower at the end of the final sentence. For the interaction between connective and plausibility, no effects were found on the Italian side, but there was a significant effect for both *evenso* (*p* < 0.05) and *however* (*p* < 0.01) connectives and plausibility on the Chinese side.

For pairwise comparisons of conditions,^7^ as shown in Table 4, the null-plausible conditions are significantly different (*p* < 0.001) from their connective counterparts and from the null-implausible condition for both languages. Furthermore, null-implausible conditions are also significantly different (*p* < 0.001) from both connective implausible conditions in both languages. There are, however, several differences between the Italian and Chinese RTs in terms of between connective conditions. Firstly, the difference between *evenso*-plausible and *however*-plausible is **not** significantly different for Italian (*p* = 1.00) but it is for Chinese (*p* < 0.001), and the same is true of the implausible counterpart (Italian: *p* = 0.93; Chinese: *p* < 0.001): in both cases, *even so* in Chinese seems to elicit significantly longer reading times. Secondly, for *evenso*-plausible and *evenso*-implausible, again there is a difference in that the Italian side is not significant (*p* = 0.98), whereas in Chinese, plausible items are read significantly faster (*p* < 0.01), although the difference is somewhat less than the other conditions. Lastly, there is no significant difference between *however*-plausible and *however*-implausible for Italian (*p* = 0.58), compared to a significant difference in Chinese (*p* < 0.01).

**Table 4:** Interaction of connective and plausibility for Italian and Chinese reading times at the end of sentence region.

**Contrast**		**Italian**			**Chinese**		
		***Est***.	**SE**	***p***	***Est***.	**SE**	***p***
Null plausible	Even-so plausible	−201.7	28.2	< 0.0001^***^	−212.1	9.21	< 0.001^***^
Null plausible	However plausible	−218.2	28.2	< 0.0001^***^	−64.92	8.54	< 0.001^***^
Null plausible	Null implausible	−88.7	23.0	< 0.01^**^	−64.20	7.22	< 0.001^***^
Null plausible	Even-so implausible	−240.7	28.2	< 0.01^**^	−246.24	9.21	< 0.001^***^
Null plausible	However implausible	−280.6	28.2	< 0.01^**^	−105.43	8.54	< 0.001^***^
Even-so plausible	However plausible	−16.5	32.6	1.0000	147.22	10.27	< 0.001^***^
Even-so plausible	Null implausible	113.00	28.2	< 0.01^**^	147.94	9.20	< 0.001^***^
Even-so plausible	Even-so implausible	−39.0	32.6	0.9808	−34.11	10.83	0.024
Even-so plausible	However implausible	−78.9	32.6	0.2114	106.71	10.27	< 0.001^***^
However plausible	Null implausible	129.5	28.2	< 0.001^***^	0.72	8.53	1.0000
However plausible	Even-so implausible	22.5	32.6	1.0000	−181.32	10.27	< 0.001^***^
However plausible	However implausible	−62.4	32.6	0.5771	−40.51	9.67	< 0.001^**^
Null implausible	Even-so implausible	−152.0	28.2	< 0.0001^***^	−182.05	9.20	< 0.001^***^
Null implausible	However implausible	−192.0	28.2	< 0.0001^***^	−41.23	8.53	< 0.001^***^
Even-so implausible	However implausible	−39.9	32.6	0.9765	140.81	10.27	< 0.001^***^

SE, standard error. Significance levels of *p* values ^*^p < 0.05, ^**^p < 0.01, ^***^p < 0.001.

Once again, the patterns observed in the two languages are slightly different. For the purpose of observing the expectation reversal in reading, particularly relevant is the comparison between the two plausibility conditions with connectives and the implausible condition without connective, since the presence of a connective can turn an otherwise implausible scenario into a plausible one. Noticeably, in Italian both *however* and *even so* plausible items are significantly different than plain implausible ones (*p* < 0.01), while in Chinese only the *even so* plausible conditions is significance (*p* < 0.001), but contrary to expectations, they are associated with *longer* reading times. We can hypothesize that, despite being plausible, conditions with connectives require additional processing time, due to more structural complexity. This marks a difference with the plausibility judgements, where participants rated the plausible connective conditions higher: this is possibly due to the fact that in the self-paced reading test, they are reading the sentence word-by-word and they have extra processing costs caused by expectation reversal caused by a connective, and this could be translated into regressions and extended reading time. But when they have to rate the semantic plausibility of the connective item as a whole, they still rate it as more plausible that the plain implausible item.

The effects of plausibility and of the *however* contrastive connective are showing up only in the end region for Chinese, but not in the target region. *Even so*, in general, seems to be associated with higher difficulty in this language compared to *however*, which might be due to the difference in frequency between the two connectives, as the Chinese *even so* (即使如此) has a logarithmic frequency of 3.21 in the combined corpora of the wordfreq tool, against 5.85 of *however* (但是).^8^

## 4 Experiments with language models

After extracting the human reading times, we compared them with the Surprisal scores of the same experimental items extracted from language models. More specifically, we used the GPT-2 Base model for both languages^9^ and the methods implemented in the Minicons library (Misra, 2022) for computing the Surprisal scores. Minicons is an open-source library that provides a high-level API for behavioral and representational analyses of NLMs. We are aware of the fact that we could have chosen different and perhaps more powerful architectures for computing our Surprisal scores. However, we initially preferred to stick with GPT-2 Base in order to select a model that has a standard implementation on HuggingFace in all the target languages, which makes the results easily comparable.

We computed the Surprisal scores for the target word in the stimuli, the word that triggers the change of a story's plausibility, as illustrated in Section 3.1 *Experimental items*. Formally, the Surprisal for the target *w*_*t*_ in the context *w*_1…*t*−1_ is defined as the negative logarithm of the probability of *w*_*t*_ given the previous context, as in Equation 1. For each target word we actually computed the summation of the Surprisals of the sub-tokens composing it, in case a word had been split by the tokenizer of the NLMs into multiple sub-tokens.


(1) $$\begin{array}{l} Surp\left(w_{t}\right)=-logP\left(w_{t}|w_{1...t-1}\right) \end{array}$$

For each dataset, we then fitted a linear mixed-effects model using the Surprisals of the target word computed by GPT-2 as the dependent variable, and the ID of each dataset item as the random intercept in our models. The independent variables include the plausibility of the discourse *Plausibility* (plausible vs. implausible), the discourse connective condition *Connective* (*EvenSo, However*, and without connective), the token length of the stimulus (*Seq_Len*), and an out-of-vocabulary (*OOV*) binary label indicating whether or not the target word in the stimulus is out of the pre-trained NLM's vocabulary. This is potentially an important factor, since NLMs do not tokenize by words but by subwords and GPT-2 makes use of a Byte-Pair (BPE) encoding tokenizer: it has been argued that the Surprisal values for words with more than one subtoken in BPE models tend to be more uniform and less cognitively realistic (Nair and Resnik, 2023). Finally, we included the interaction between the Connective and the Plausibility conditions.

Once again, we used the *lmerTest* package (Kuznetsova et al., 2017) for generalized linear mixed model fitting and results, as illustrated in Table 5. As a difference from the models for the reading times data, in this case we only have random effects for the items, but not for participants (all the predictions for a set of data in one language come from a single language model).

**Table 5:** Summary for the linear-mixed effects models results of predictors of Surprisals with the *Italian* and *Chinese* datasets.

	**Italian**			**Chinese**		
	***Est***.	**SE**	***p***	***Est***.	**SE**	***p***
Intercept	8.54	3.43	< 0.05^*^	22.87	4.70	< 0.001^***^
ConnectiveE	−0.21	0.75	0.78	1.45	0.29	< 0.001^***^
ConnectiveH	0.28	0.72	0.70	1.10	0.16	< 0.001^***^
Implausible	0.29	0.72	0.68	0.70	0.13	< 0.001^***^
OOV	4.06	0.72	< 0.001^***^	−1.82	0.59	< 0.01^**^
Seq_Len	0.26	0.09	< 0.01^**^	−0.16	0.12	0.16
Freq	−1.97	0.44	< 0.001^***^	−1.70	0.39	< 0.001^***^
ConnectiveE:implausible	0.21	1.02	0.84	−0.96	0.23	0.06
ConnectiveH:implausible	0.34	1.02	0.74	−0.43	0.23	< 0.001^***^

ConnectiveE, Discourse with the connective “Even so”; ConnectiveH, Discourse with the connective “However”; PlausibilityP, a plausible discourse; Seq_Len, sequence length, namely the number of tokens in the sequence; out-of-vocabulary label OOV indicating whether the word is in the NLM's pre-training vocabulary (0) or not (1), SE, standard error. Significance levels of *p* values ^*^p < 0.05, ^**^p < 0.01, ^***^p < 0.001.

**Figure 3:**
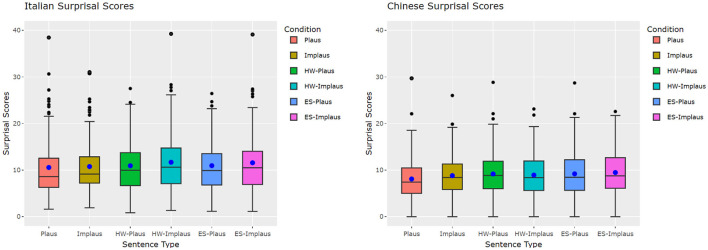
Boxplots of the NLM Surprisals for discourse connectives. Inner box dot, mean; inner box line, median; outer box dots, outliers. Plaus, plausible without connective; Implaus, implausible without connective; HW-(Im)Plaus, (Im)plausible with *however*; ES-(Im)Plaus, (Im)plausible with *even so*.

We visualized NLMs' Surprisal scores distribution in the conditions for both the Italian and the Chinese datasets in Figure 3. Observing the boxplots suggests that the plausible without-connective condition leads to the lowest overall Surprisal scores across languages and connective types. There are observably more outliers in the Italian datasets than in the Chinese datasets. The data distribution is more “normal” in the Chinese datasets than in the Italian datasets, as suggested by the smaller gaps between mean and median.

For linear mixed-effects models, in the Italian dataset, Table 5 shows that there is no plausibility effect in the Surprisal scores, while there is a strongly significant effect for Seq_Len and a significant effect for OOV: the increase of both variables leads to an increase in Surprisal scores. There is also a significant effect with the frequency of the target word: more frequent words are associated with the decrease of the Surprisal. The interactions of the predictor variables did not give rise to any significant effect.

On the other hand, in Chinese, all the predictors except for Seq_Len can be seen to have a significant effect on the scores: implausible items are associated with an increase in Surprisal, as well as both the Connective conditions. Increase of frequency and out-of-vocabulary words are associated instead with a Surprisal decrease. While the latter might seem surprising, it should be pointed out that the vocabulary of Chinese LLMs has characters as main units instead of words, so that unless the target word is one-character long, OOV will be a default condition for the Chinese data: if we look at the vocabulary of the two GPT-2 models, we can notice that the 77% of the items in Chinese have OOV target words, against 45% in the Italian dataset. Given that the Surprisal is estimated at the character level and then calculated by summing the scores of the characters composing a word, it is in theory possible to observe OOV, multi-character words with relatively low Surprisals if the characters composing them are all highly predictable. We will dedicate an additional analysis to shed further light on this observation.

While interactions in Italian were not significant, *However* turned out to interact significantly with plausibility in Chinese (*ps* < 0.001). The interaction with *even so* was not significant (*p*>0.05), as a difference from what was observed in the human data.

Looking then at the pairwise comparison scores by plausibility condition in Table 6, we can observe that:

- almost no significant differences can be observed in the Italian data, except for the *however* implausible items being more surprising than *even so* ones;
- among Chinese plausible items, plain items are significantly less surprising than both connective conditions at *p* < 0.0001;
- among Chinese items with no connectives, implausible items are significantly more surprising than plausible ones (*p* < 0.0001);
- Chinese *even so* implausible items are more surprising than plain implausible ones (*p* < 0.01);
- in both languages, there is no difference between the implausible no connective condition and the plausible ones with connectives.

**Table 6:** Interaction of connective and plausibility for Italian and Chinese LMs.

**Contrast**		**Italian**			**Chinese**		
		***Est***.	**SE**	***p***	***Est***.	**SE**	***p***
Null plausible	However plausible	−0.28	0.72	1.00	−1.10	0.16	< 0.0001^***^
Null plausible	Even-so plausible	0.21	0.75	1.00	−1.45	0.29	< 0.0001^***^
Null plausible	Null implausible	−0.29	0.72	1.00	−0.70	0.13	< 0.0001^***^
Null plausible	However implausible	−0.92	0.72	0.97	−0.84	0.17	< 0.0001^***^
Null plausible	Even-so implausible	−0.29	0.76	1.00	−0.72	0.30	< 0.0001^***^
However plausible	Even-so plausible	0.50	0.18	0.09	−0.35	0.30	0.98
However plausible	Null implausible	0.50	0.18	0.09	0.40	0.16	0.21
However plausible	However implausible	−0.64	0.72	1.00	0.26	0.19	0.94
However plausible	Even-so implausible	−0.01	0.75	1.00	−0.62	0.30	0.48
Even-so plausible	Null implausible	−0.51	0.74	1.00	0.74	0.29	0.14
Even-so plausible	However implausible	−1.13	0.72	0.86	0.61	0.30	0.45
Even-so plausible	Even-so implausible	−0.51	0.72	1.00	−0.26	0.19	0.92
Null implausible	However implausible	−0.63	0.73	1.00	−0.14	0.17	1.00
Null implausible	Even-so implausible	0.00	0.77	1.00	−1.02	0.30	< 0.01^**^
However implausible	Even-so implausible	0.63	0.18	< 0.01^**^	−0.88	0.30	0.05

SE, standard error. Significance levels of *p* values ^*^p < 0.05, ^**^p < 0.01, ^***^p < 0.001.

If we compare the human experiments and the NLMs, we observe that the effects found with Chinese NLMs are similar to the ones in human data: they are sensitive to plausibility effects and they assign lower Surprisals to plausible items, although this effect shows up already at the target word in GPT-2 and only in the end region in the human reading task. Chinese NLMs are aligned with humans also in the sense that both connective conditions lead to an increase in reading times in humans and to higher Surprisal scores in GPT-2. On the other hand, there was a significant interaction between Connective and Plausibility in the human data, while in Chinese NLMs the effect is stronger with *however* and it does not reach significance for *even so*. Finally, despite the similarity in the effects with humans, none of the models showed the expectation reversal comparing the plain implausible conditions with the plausible ones with connectives. This means that, although NLMs' predictions are affected by sentence plausibility and connectives, the Surprisal scores at the target word do not reflect any connective-related shift in the predictability of an implausible target word.

The NLM results in Italian instead are not at all aligned with human data. Unlike humans, who read plausible items faster, NLM scores show no plausibility effect. This is an unexpected result, as previous work showed that English NLMs are generally good at distinguishing between plausible and implausible sentences (Kauf et al., 2022; Hu and Levy, 2023; Amouyal et al., 2024; Kauf et al., 2024). Moreover, there is no trace of significant effects of the connective in the Surprisal scores of NLMs, while they were shown to add more complexity in human reading. We discuss possible reasons for misalignment between humans and NLMs in the following subsection.

### 4.1 Results analysis and discussion

In our experiments with NLMs, we used two language-specific GPT-2 models to compute the Surprisals scores at the target verb and assess the extent to which the predicted pattern resembles the one observed in human readers. This is similar to the study by Cong et al. (2023b) on English, although we only used GPT-2 Base as the largest autoregressive model that was available for both Italian and Chinese at the time of the initial experiments. To our knowledge, different GPT sizes are not available as open models for these two languages.

In Chinese, the NLMs reproduced the effects of Plausibility and Connectives and their direction (implausible items and connectives lead to a significant increase in reading times), although it should be pointed out that (i) the NLM Surprisal scores were computed at the target verb region, while the corresponding effects in Chinese emerged only in the post-target region; (ii) there was a significant interaction between Connective and Plausibility that was absent in the human data; (iii) no expectation reversal effects are found in the comparisons plain implausible vs. connective plausible items. On the other hand, the NLM results for Italian showed a very weak alignment with human behavior, since (i) no plausibility effect was found in the Surprisal scores; (ii) the only connective-related effect was found for *even so*, and in the opposite direction to the one observed in the self-paced reading task (a decreases of the Surprisal of the NLM vs. the increase of human reading times). What are the possible reasons for this negative result in Italian?

One possibility is that, concerning the models used in Cong et al. (2023b), our GPT-2 Base is not powerful enough to model the datasets. The Surprisal extracted from larger models with lower perplexity might have a higher predictive power of the reading times (Goodkind and Bicknell, 2018; Hao et al., 2020; Wilcox et al., 2020),^10^ and indeed Cong et al. (2023b) obtained their closest alignment results with a GPT-Neo model (1.3 B parameters, vs. 124 M for GPT-2 Base).

Another possibility is that there is a major problem in the limited coverage of the target words in the model vocabulary: while in Cong et al. (2023b) only 14% of the targets were split by the BPE tokenizer, in the Chinese and the Italian datasets we have much higher percentages of OOV target words (77 and 45%, respectively). The work by Nair and Resnik (2023) recently showed that the estimation of Surprisal for words composed of multiple subtokens in English can be problematic, and it is reasonable to assume that the issue would extend also to other languages. On top of that, and possibly still related to the splitting of OOV words into multiple subtokens, it should be noted that in our data the effects of the sequence length have a larger magnitude than in the concessive experiment of Cong et al. (2023b). These two possible explanations are, of course, not mutually exclusive - both factors might have concurred to produce our negative result with NLMs.

To test the first possibility, we repeat our NLMs experiment with the recently-introduced Llama-2-7B model, a large autoregressive architecture with that is available for both Chinese and Italian. For Chinese, we use the implementation by Cui et al. (2023), while for Italian we use the one by Basile et al. (2023) (“Llamantino”). The procedure for extracting Surprisals and the setup of the linear mixed effects models are exactly the same. The results are displayed in Table 7.

**Table 7:** Summary for the linear-mixed effects models results of predictors of Llama-2 model Surprisals.

	**Italian**			**Chinese**		
	***Est***.	**SE**	***p***	***Est***.	**SE**	***p***
Intercept	3.23	5.10	0.53	11.39	2.87	< 0.001^***^
ConnectiveE	−0.02	1.14	0.98	1.52	0.19	< 0.001^***^
ConnectiveH	0.98	1.10	0.38	1.82	0.18	< 0.001^***^
Implausible	1.66	1.10	0.13	1.30	0.15	< 0.001^***^
OOV	2.88	1.99	0.15	−2.52	0.65	< 0.001^***^
Seq_Len	0.24	0.09	< 0.01^**^	0.09	0.08	0.24
Freq	−1.71	0.52	< 0.01^**^	−1.59	0.40	< 0.001^***^
ConnectiveE:implausible	0.44	1.56	0.78	−1.80	0.25	< 0.001^***^
ConnectiveH:implausible	0.64	1.02	0.68	−1.77	0.25	< 0.001^***^

SE, standard error. Significance levels of *p* values ^*^p < 0.05, ^**^p < 0.01, ^***^p < 0.001.

It can be immediately seen that the pattern for Chinese is very similar, with similar effects. Now both the interactions between connective and plausibility are significant, but the pairwise comparisons still do not reveal any significant difference between the expectation reversal conditions (*p*>0.05 for null implausible vs. *even so* plausible and null implausible vs. *however* plausible). Interestingly, the result that out of vocabulary words are associated with lower Surprisals is consistent. On the other hand, the situation is mostly unchanged for Italian: the only significant effects are for sequence length and frequency. Given that the Llama-2 model is much larger than previously-employed NLMs for Surprisal estimation (e.g., 7B size against a max size of 1.3B for the models employed by Cong et al., 2023a), it does not look like the cause of the misalignment was the Italian NLMs being too small or not powerful enough.

The difficulty of the Italian NLM might be related to tokenization issues, as implied by recent studies such as Nair and Resnik (2023): since many of the target words (45% of targets for Italian) are out-of-vocabulary, it is possible that the model is unable to provide accurate Surprisal scores simply by summing the individual scores of the subtokens.

To verify the relationship between OOVs and inaccurate predictions in our data, we set up a simple linear model using the z-scores of the GPT-2 Surprisal distribution as the target variable. Z-scores turn each data point into its distance in terms of number of standard deviations from the mean of the distribution, and data points with high positive values can be considered as outliers—or, otherwise said, words that were very surprising for our language models. Notice that in our Surprisals distribution we only have positive outliers, as all the points with lower values than the mean are within two standard deviations (Italian: Min = −1.63, Max = 4.56; Chinese: Min = −1.86, Max = 4.41). As predictors for the z-scores, we simply use the logarithmic frequency and the OOV variable. We are especially interested in seing whether OOV words determine a significant increase in z-scores.

The results can be seen in Table 8, showing a clear contrast between the two languages. It can be seen indeed that while there is no effect of OOVs on the z-scores of the Chinese Surprisals, those are associated with a significant increase in the z-scores in the Italian data (*p* < 0.001). This seems to confirm the issue raised by Nair and Resnik (2023), which pointed out the potential problem with OOV probability estimation with NLMs based on the BPE tokenizer, and proposed the use of morphologically-aware tokenizers as a more cognitively-plausible alternative. It is striking that in Chinese, although the percentage of OOV targets is even higher than in Italian, this effect is not observed. We hypothesized that is due to the peculiarity of character-based languages, where most of the target words would be OOV for NLMs with BPE tokenizers. Our assumption is that, in Chinese, multi-character words can be composed by easy-to-predict characters with strong statistical associations between each other, such that the Surprisal of the word as a whole will not be significantly higher than single-character words.

**Table 8:** Surprisal *z*-score predictors in Italian and Chinese.

	**Italian**			**Chinese**		
	***Est***.	**SE**	***p***	***Est***.	**SE**	***p***
Intercept	0.02	0.46	0.97	1.48	1.02	0.15
OOV	2.28	1.14	< 0.001^***^	0.00	1.07	1.00
Freq	−0.11	0.10	0.27	−0.28	0.22	0.20
OOV*freq	−0.41	0.14	0.00	0.09	0.23	0.70

SE, standard error. Significance levels of *p* values ^*^p < 0.05, ^**^p < 0.01, ^***^p < 0.001.

## 5 Conclusions

In this paper, we investigated how discourse connectives affect the predictions of upcoming events (i.e., the target verbs) in human sentence processing and NLMs in Italian and Chinese. We focused on concessive and contrastive connectives, that can be used to reverse the event-based expectations of the subjects.

We observed some interesting differences in both the ratings and the self-paced reading times of Italian and Chinese speakers. Italian speakers showed clear plausibility distinctions, rating the plausible condition much higher than the implausible one in the corresponding connective condition, and without big differences between implausible items. In Chinese, we observe that speakers rate the no connective implausible conditions as the least plausible of all the connective conditions, and that the connectives' effect is to reduce differences in plausibility. In the self-paced reading experiment, we noticed significantly increased reading times in Italian for implausible items and items with connectives, both in the target and in the post-target region. In Chinese, there was an effect of slower reading times with *even so* in the target region, but the same effects found in the Italian group emerged later in the Chinese group in the region of the last word of the sentence. We did not observe striking processing differences between connectives in Italian, when comparing items of similar plausibility; on the other hand, in Chinese the concessive seems to be a more rare structure and it elicits significantly longer reading times.

The comparisons between an implausible condition without connective and the corresponding plausible conditions with a connective reveal that the latter generally elicit significantly longer reading times (with the only exception of the Chinese *however*), probably due to an increased structural complexity in the sentence, although the plausibility ratings of the speakers tend to be higher for plausible conditions. This suggests that the expectation reversal increases processing complexity, on the one hand, but on the other hand humans judge the reversed situations as more plausible, when given some extra time after reading the final sentence.

In our experiments with NLMs, a GPT-2 model for Chinese reproduced most of the main effects (plausibility and connectives) that were observed at the end of the sentence region in human reading data, whereas a similar model for Italian was totally misaligned, showing none of the above-mentioned effects. Although the Chinese models are closer to human results in terms of main effects (and also in terms of interactions, in the case of Llama-2), however, their alignment is far from perfect, because none of them assigns significantly different scores to the expectation reversal conditions.

For the clear misalignment between human and NLMs in Italian, we finally advanced two possible explanations: the relatively small size of the GPT-2, which might not be powerful enough to account for the differences in the data; or a general difficulty of NLMs with BPE tokenizers in estimating Surprisals for morphology-rich languages. To test the first possibility, we re-ran the experiments using a more powerful model, Llama-2, to compute Surprisals but the results in Italian did not change. On the other hand, we tested whether out-of-vocabulary words are associated with larger *z*-scores for Surprisal and we observed that this is the case for Italian, but not for Chinese, thus providing support for the second explanation.

In conclusion, our results encourage us to run experiments on sentence processing and NLMs on multiple languages, to compare human behavior in a crosslinguistic fashion, and to try to reproduce NLM results, since not all languages have the same models and resources that are available in the English language. Future work in the field will also have to take into account the issue of the tokenizers, as BPE encoding might not be the most suitable solution for computing the Surprisals of OOV words: morphologically-aware tokenizers might be a necessary solution to account for different notions of word in different languages (Nair and Resnik, 2023) and obtain realistic estimates for rarer words.

## Data Availability

The datasets presented in this study can be found in online repositories. The names of the repository/repositories and accession number(s) can be found below: https://github.com/yancong222/LMs-discourse-connectives-Surprisals.

## Footnotes

^1^An ERP (event-related potential) is an electrophysiological response of the brain to a stimulus and the N400, one of the most commonly studied ERP components, is a negative-going deflection that peaks around 400 milliseconds after presentation of the stimulus. Although there are different interpretations of its meaning, there is a general agreement among researchers that it may represent a sort of brain signature of semantic complexity (Hagoort, 2003). A larger N400 is thus interpreted as an index of an increased semantic complexity, while a reduced N400 corresponds to a facilitation effect.

^2^There are actually two main classes of NLMs: the *autoregressive* models (e.g., the members of the GPT family) are unidirectional and predict the probability of the next word given the previous context; the *denoising autoencoding* models (e.g., BERT, RoBERTa etc.) are bidirectional and are trained on a masked language modeling task, where some random words in an input sentence are replaced by a [MASK] token and the goal for the network is to predict the original words on the basis of the left and right context. In this paper, we will refer to the class of autoregressive language models, because their unidirectional nature makes them a more plausible choice for modeling human reading behavior.

^3^We talk about facilitation effects when the context in which a word is encountered facilitates its processing, as it can be observed from behavioral (e.g., shorter reading times or eye fixations) or neurophysiological indicators (e.g., a reduced N400 amplitude). On the other hand, interference effects occur when readers incorrectly retrieve from their working memory sentence elements that are similar to those required during the comprehension processes (Tan et al., 2017).

^4^GPT-2 (Radford et al., 2019), a variation of GPT, is a uni-directional. Transformer-based language model, which means that it has been trained on predicting the next word, given all of the previous words. It is commonly used in computational psycholinguistics for modeling reading times data.

^5^ https://www.deepl.com/en/translator

^6^ https://github.com/rspeer/wordfreq

^7^For cond1:cond2 the reference level is the first/the second condition, therefore a positive/negative estimate means an increase/decrease from cond1/cond2 to cond1/cond2.

^8^wordfreq frequencies have been extracted from a combination of corpora from the SUBTLEX project (Brysbaert and New, 2009).

^9^Italian model (de Vries and Nissim, 2021): https://huggingface.co/GroNLP/gpt2-small-italian; Chinese model (Zhao et al., 2019): https://huggingface.co/uer/gpt2-chinese-cluecorpussmall.

^10^However, see also the results of Oh and Schuler (2023): the Surprisal of larger NLMs does not always provide a better fit to human reading times, especially because larger models tend to achieve a superhuman performance on the prediction of open class words (e.g., nouns, adjectives).
